# Micronutrients and cognitive functions among urban school-going children and adolescents: A cross-sectional multicentric study from India

**DOI:** 10.1371/journal.pone.0281247

**Published:** 2023-02-02

**Authors:** Shweta Singh, Shally Awasthi, Divas Kumar, Seema Rani Sarraf, Anuj Kumar Pandey, Girdhar G. Agarwal, Avivar Awasthi, Anish T. S., Joseph L. Mathew, Sonali Kar, Suma Nair, Chythra R. Rao, Harsh Pande, B. N. Mahanta, Bhavneet Bharti, C. M. Singh, Kuldeep Singh, Mushtaq A. Bhat, Somashekar A. R., Rajiv Awasthi, Abbas Ali Mahdi

**Affiliations:** 1 Department of Psychiatry, King George’s Medical University, Lucknow, Uttar Pradesh, India; 2 Department of Pediatrics, King George’s Medical University, Lucknow, Uttar Pradesh, India; 3 Department of Statistics, University of Lucknow, Lucknow, Uttar Pradesh, India; 4 Department of Endocrinology, Medical College, Kolkata, India; 5 Department of Community Medicine, Government Medical College, Thiruvananthapuram, Kerela, India; 6 Department of Pediatric Medicine, Post Graduate Institute of Medical Sciences, Chandigarh, India; 7 Department of Community Medicine, Kalinga Institute of Medical Sciences, Bhubaneswar, Orissa, India; 8 Department of Community Medicine, Kasturba Medical College, Manipal, Karnataka, India; 9 Department of Medicine, Assam Medical College, Dibrugarh, Assam, India; 10 Department of Community & Family Medicine, All India Institute of Medical Sciences, Patna, Bihar, India; 11 Department of Pediatrics, All India Institute of Medical Sciences, Jodhpur, Rajasthan, India; 12 Department of Pediatrics, Sher-i-Kashmir Institute of Medical Sciences, Srinagar, Jammu & Kashmir, India; 13 Department of Pediatrics, M. S. Ramaiah Institute of Medical Sciences, Bangalore, Karnataka, India; 14 Prarthana Diabetic Care Centre, Lucknow, Uttar Pradesh, India; 15 Department of Biochemistry, King George’s Medical University, Lucknow, Uttar Pradesh, India; Jazan University, SAUDI ARABIA

## Abstract

**Background:**

Micronutrient deficiency (MD) is associated with deficits in cognitive functioning of children. However, no comprehensive multicentric study has been conducted in India to explore the role of multiple MD in cognition of children and adolescents. The present study aimed to explore association of MD with level of general intelligence and specific cognitive functions, in urban school-going children and adolescents across ten cities of India.

**Method:**

Cross-sectional multicentric study, enrolled participants aged 6–16 years. Blood samples were collected for biochemical analysis of calcium, iron, zinc, selenium, folate, vitamin A, D and B12. Colored Progressive Matrices / Standard Progressive Matrices (CPM/SPM), Coding, Digit Span and Arithmetic tests were used for the assessment of cognitive functions of participants. Height and weight measures were collected along with socio-economic status.

**Results:**

From April-2019 to February-2020, 2428 participants were recruited from 60 schools. No MD was found in 7.0% (134/1918), any one MD in 23.8% (457/1918) and ≥ 2 MD in 69.2% (1327/1918) participants. In presence of ≥ 2 MD, adjusted odds ratio (OR) for borderline or dull normal in CPM/SPM was 1.63, (95% CI: 1.05–2.52), coding was 1.66 (95% CI: 1.02–2.71), digit span was 1.55 (95% CI: 1.06–2.25) and arithmetic was 1.72 (95% CI: 1.17–2.53), controlling for gender, socioeconomic status and anthropometric indicators.

**Conclusion:**

Since ≥ 2 MD were found in more than 2/3rd of participants and was associated with impairment in cognitive function, attempts must be made to ameliorate them on priority in school going children in India.

**Trial registration number:**

CTRI/2019/02/017783.

## Introduction

Micronutrients are vital components of diet that are required for a wide range of physiological and cognitive functions. Being integral to various metabolic pathways in the brain, the deficiency of a micronutrient can potentially impact the development and functioning of the nervous system adversely. Due to this, every year, millions of children are affected not only from physiological challenges (stunted growth, weakened immunity and physical diseases) but also cognitive and intellectual deficits [1–4]. Studies demonstrate that deficiency of micronutrients is associated with children’s impaired cognitive development and poor classroom performance [5–7].

Various micronutrients and hemoglobin have a major role in physical and cognitive development. Poor cognitive development, loss of concentration and even lower intelligence has been reported with deficiencies of iron [8], vitamin B12 [9, 10] and folate [11, 12]. Impaired behavior and cognition have also been reported with deficiency of zinc [13] and vitamin D [14].

Studies conducted in India, among school aged children and adolescents, reported widespread deficiency of micronutrients [7, 15–19]. However, no comprehensive multicentric study has been conducted in India to assess the impact of various crucial micronutrient deficiencies on general intelligence as well as specific cognitive functions in children and adolescents. The present study set out to explore association of micronutrient deficiency with level of general intelligence, and specific cognitive functions, i.e. attention, concentration, visuomotor coordination and working memory, in urban school-going children and adolescents aged 6 to 16 years across ten cities of India. The primary objective was to assess the association of no micronutrient deficiency, any one or ≥2 micronutrient deficiencies with cognitive performance as determined by Colored Progressive Matrices (CPM) / Standard Progressive Matrices (SPM), coding, digit span and arithmetic tests. The secondary objective was to assess association of cognitive performance, as determined by CPM/SPM, coding, digit span and arithmetic tests, with micronutrient deficiencies controlling for socioeconomic variables.

## Methodology

### Ethical approval and consent to participate

The study was approved by the Institutional Ethics Committee for MS Ramaiah Medical College and Hospital Bangalore (approval reference number (ARN): MSRMC/EC/AP-02/02-2019), Institutional Ethics Committee for Kalinga Institute of Medical Sciences Bhubaneswar (ARN: KIMS/KIIT/IEC/112/2016), Institutional Ethics Committee for PGIMER Chandigarh (ARN: PGI/IEC/2019/000152), Institutional Ethics Committee (H) Assam Medical College (ARN: AMC/EC/1430), Institutional Ethics Committee for All India Institute of Medical Sciences Jodhpur (ARN: AIIMS/IEC/2017/765), Institutional Ethics Committee for King Georges Medical University (ARN: 9334/Ethics/R.Cell-16), Institutional Ethics Committee for Kasturba Medical College (ARN: IEC:388/2019), Institutional Ethics Committee for All India Institute of Medical Sciences Patna (ARN: IEC/AIIMS/PAT/153/2017), Institutional Ethics Committee for Sher-i-Kashmir Institute of Medical Sciences (ARN: IEC/SKIMS Protocol # RP 175/2018) and Human Ethics Committee Medical College Thiruvananthapuram (ARN: HEC.No.04/34/2019/MCT). The study was registered prospectively with Clinical Trial Registry of India (registration number CTRI/2019/02/017783). Written informed consent was obtained from parents of all study participants.

### Study design and setting

This cross-sectional multicentric study was conducted across ten cities of India viz Bangalore, Bhubaneswar, Chandigarh, Dibrugarh, Jodhpur, Lucknow, Udupi (Manipal), Patna, Srinagar and Thiruvananthapuram, centrally coordinated by King George’s Medical University, Lucknow (KGMU Lucknow). The detailed study protocol is published elsewhere [20].

### Sample size computation

The prevalence of folate deficiency in India approximately is 30.7% [21], precision (d) of 2% and level of significance (α) 0.05, the calculated sample size [22] was 2044 participants. After taking, attrition rate of 10% sample size inflate to 2400 participants. This sample size was equally divided among the ten cities. Sample size was calculated using folate deficiency as this gave the maximum sample.

### Selection of participants

Participants were selected by using a two-stage sampling technique. In the first stage, schools were selected, and in the second stage, participants were recruited from the selected schools. Each study site provided a list of government as well as private schools enrolling children (both girls and boys) between 6 to 16 years of age and located within the city’s urban limits. From this list, six schools were selected using simple random sampling, having at least one to a maximum of three private schools. Principals of the recruited schools were met to obtain voluntary written informed consent for the school’s participation. Then, with the help of an identified coordinating school teacher, a gender-wise list of students between 6 to 11 (group 1) and 12 to 16 years (group 2) of age was prepared and numbered serially. From each of these lists, first fifteen healthy students, residing within five kilometres of the school were selected by random number draw. They were invited to participate in the study. The first ten students whose parents provided written informed consent for participation, were included in the study. The rest were kept as reserve. Written assent was obtained from all children older than 8 years. Those having BMI less than 12.5 were excluded from the study, and their parents were informed and advised to seek medical consultation.

### Tools for cognitive assessment

All the participants were assessed for general intelligence and specific cognitive functions.

*General Intelligence*: Standard Progressive Matrices (SPM) [23] and Colored Progressive Matrices (CPM) [24] were used to assess the general intelligence of participants. These two matrices are part of a series of Raven’s Progressive Matrices (RPM) developed by John C. Raven (1936) [23, 24]. RPM is a multiple-choice intelligence test of abstract reasoning. In each test item, the subject needs to choose the correct missing piece that completes a pattern. SPM was published for children aged 11 years or above. The internal consistency reliability estimates and split-half reliability for SPM were 0.88 and 0.84, respectively. CPM is designed for younger children (between 6 to 11 years of age). Test-retest reliability was reported at a value of 0.86, while validity ranged from 0.60 to 0.70 for Indian children.

*Specific cognitive functions*: To assess specific cognitive functions, subtests of Malin’s Intelligence Scale for Indian Children (MISIC) [25] were used. MISIC is an Indian adaptation of ‘Wechsler Intelligence Scale for Children’, an American test developed by Dr David Wechsler which is used for children of ages 6 years and above and administered individually [25]. Tests used were as follows:

1. *Attention and concentration*: These were assessed using Digit Span Test, which involves a verbal narration of a sequence of numbers to the subject and a subsequent repetition of the numbers in the same sequence by the subject to the test-taker. Digit Span is traditionally done in two parts: Digit Span Forward and Digit Span backward. The total score for the Digit Span Test is the sum of forwards and backwards test scores.
2. *Visual-spatial ability*: Coding Test was used to assess visual-spatial ability. This test consists of certain symbols that are paired with numbers or shapes. The subject has got to learn them and pair them with the suitable corresponding number or shape. This test requires concentration and speed. The test is divided into two parts, coding A and coding B. Coding A is for subjects below 8 years of age or those with suspected mental defects. Coding B is for subjects above 8 years of age. The time limit for test completion is 120 seconds. The score depends on the number of designs completed within the above time limit (excluding samples). A bonus point is added if the subject completes the test before the set time, while one point is given for every correct response.
3. *Working memory*: Working memory was assessed using arithmetic test. It contains twenty simple mental mathematical calculations similar to problems of elementary school level. Problems are administered orally and must be solved without paper or pencil. After stating the problem, the timer starts. If the child asks to repeat the question, then repetitions are at the expense of their timing. After three consecutive failures, the test is discontinued. Scores are awarded in 1s or 0s. For subjects above 8 years of age, problem number four is administered directly. If they correctly answer problem number 4 and 5, credit points are given for problem numbers 1 to 3.

The reliability of MISIC was established with the test-retest method, and it yielded a Pearson’s Product Moment correlation coefficient of 0.91 for full-scale intelligent quotient (IQ) result. MISIC established concurrent validity from school ranking, whereas congruent validity for the upper age level was established from the California short-form test of Mental Maturity (an adapted version) and for the lower age level from the Good Enough Draw a Man test. Both yielded a coefficient of 0.63. IQs were obtained using Indian norms based on percentile points, converted through the Thomson formula.

### Training of study team

To conduct cognitive assessment, a psychologist was appointed at each site, who worked along with other team members. They were trained by SA and SS, to administer psychological tests and document then results, at KGMU Lucknow between March to July 2019. Cognitive assessment tools were given to each site. In addition, common training on study protocol, procedures, data collection methods and instruments, was done by SA.

### Data collection

The data was collected from April 2019 to Feb 2020.

#### Demographic and socioeconomic data

Demographic and socioeconomic details were recorded by interviewing participants and their primary care giver. Revised Kuppuswamy’s socioeconomic scale [26] was used to assess socioeconomic status. This scale assess the socioeconomic status based on monthly family income, occupation and education of head of family.

#### Anthropometric measurements

Anthropometry was done by qualified and trained nutritionists. The height was measured to the nearest 0.1 centimeter using Seca 213 Mobile Stadiometer (Seca, Hamburg, Deutschland). Two measurements of height were recorded for each participant. If the difference between the two height measurements was greater than 5 mm, then a second set of two height measurements was taken to obtain more precise values. Weight was measured to the nearest 0.1 kilogram using portable Seca 803 weighing scale (Seca, Hamburg, Deutschland). The unit was standardized by calibrating it to zero before each measurement.

Body mass index (BMI) was calculated using the standard equation:

$$\text{BMI}\;\left(\frac{\text{kg}}{\text{m2}}\right)=\text{Weight}\;\left(\text{in}\;\text{kg}\right)/{\left[\text{Height}\;\left(\text{in}\;\text{m}\right)\right]}^{2}$$

We categorised the participants as severely thin, thin, normal, overweight, obese and severely obese based on their BMI for age as recommended by WHO expert committee for assessing anthropometric indicators [27].

#### Cognitive assessment

The cognitive assessment tools were administered to the subjects in individual sessions. Instructions were followed according to the test manual. The sequence of tests administrated was CPM/SPM, coding test, arithmetic test and digit span test. For those who could not complete the tests in a single session, another session was planned at their convenience. Each participant took about 40 to 50 minutes to complete the battery of tests and four to five participants were assessed daily. Variables like time taken by the participant, total score, standard score and interpretation, were entered on case record form (CRF).

#### Blood sample collection

Blood sampling was done at school by trained phlebotomists during school hours. A venous blood sample of 6 ml (4 ml in clot activator and 2 ml in EDTA vial) was collected using vacuum-tube systems, preferably from the cubital vein. All aseptic precautions were taken. Participants were kept under observation for 15 minutes after sample collection.

During the transportation of blood samples from school to study sites, the temperature was maintained between 2°C to 8°C, using pre-frozen gel packs in an ice box. Within two hours, these samples were centrifuged at 1500 rpm for 10 minutes at 4°C to separate plasma, serum and packed cells at the study site. Plasma and serum were stored below -20°C in trace element-free cyro tubes and packed cells between 2°C to 8°C, at the study sites. Samples from study sites to KGMU Lucknow were transported in two batches of 120 each, maintaining the temperature below -20°C for plasma and serum and between 2°C to 8°C for packed cells, by professional agencies having expertise in handling and shipment of bio-medical samples. Samples were prevented from exposure to light during the whole process.

### Biochemical analysis

Blood samples were analyzed at the KGMU Lucknow, to access the levels of calcium, iron, zin, selenium, folate, vitamin A, vitamin D and vitamin B12.

Quantitative determination of zinc and selenium in the serum was done by Inductively Coupled Plasma-Optical Emission Spectrometer (ICP-OES, Optima 8000, Perkin Elmer, USA). The level of vitamin D, Vitamin B12 and folate were analyzed in serum by Chemiluminescent Microparticle Immunoassay (CMIA) technology with flexible assay protocol provided by kit ARCHITECT 25-OH VITAMIN D, ARCHITECT B12 and ARCHITECT Folate (Abbot Diagnostics, Iceland) respectively. Serum calcium and iron were measured by fully automatic analyzer by Selectra PRO M. Calcium and Iron kits were provided by calcium arsenazo III colorimetric Labkit, Delhi, India and ELITGroup empowering IVD, USA, respectively. Vitamin A was estimated in serum using ELISA (Enzyme Linked Immunosorbent Assay) method according to the manufacturer’s recommendation (USCN Wuhan USCN Business Co., Ltd. cat# CED051Ge). The detailed method of biochemical analysis is published elsewhere [20].

Micronutrient deficiencies were estimated using the cut off levels as given in Table 1 [29–37]. Anemia was defined on basis of hemoglobin was estimated using the WHO defined age and gender specific cut-off [28] (Table 1).

**Table 1:** Deficiency cut off levels of micronutrients and hemoglobin.

	Age group	Gender	Cut off levels	Reference
Calcium	6 to 16 years	male & female	<10 mg/dl	[29, 30]
Iron			<70 μg/dl	[31, 32]
Selenium			< 5.5 μg/dl	[33]
Zinc	< 10 years	male & female	<65 μg/dl	[34]
	≥ 10 years	Male	<70 μg/dl	
	≥ 10 years	female	<66 μg/dl	
Vitamin A	6 to 16 years	male & female	<20.0 μg/dl	[35]
25 Hydroxy Vitamin D			<12 ng/ml	[36]
Folate			<3 ng/ml	[37]
Vitamin B12			<203 pg/ml	[36]
Mild-Anemia (Hemoglobin levels)	6 to 11 years	male & female	11.0 to 11.4 g/dl	[28]
	12 to 14 years	Male	11.0 to 11.9 g/dl	
	12 to 16 years	female	11.0 to 11.9 g/dl	
	15 to 16 years	male	11.0 to 12.9 g/dl	
Moderate-Anemia (Hemoglobin levels)	6 to 16 years	male & female	8.0 to 10.9	
Severe-Anemia (Hemoglobin levels)	6 to 16 years	male & female	< 8.0	

### Quality assurance

Data from all the sites were sent monthly through email, and the entire data was maintained and analyzed at KGMU Lucknow. Robust mechanisms were employed to maintain the quality of data collection. Data collection at sites was done under the direct supervision of the Site Investigator / Co-Investigator. KGMU, Lucknow monitored data quality by onsite monitoring to observe the data collection process and ascertain this had been done as per standard operating procedures. Scoring sheets for cognitive assessments were assessed to score and interpret results at KGMU, Lucknow. Retraining was imparted where lacunae were identified. Internal and external quality control of blood sample analysis was maintained by running controls along with the samples and participating in external quality assurance programs.

### Statistical analysis

Double data entry was done in MS excel. Data was matched electronically, and discrepancies were rectified by referring to the source documents.

Cognitive tests were categorized as into five categories as “borderline”, “dull normal”, “average”, “above average” and “superior”. For the purpose of analysis, borderline and dull normal were combined to one category and average, above average, superior was combined to another category. Cognitive tests results were treated as dichotomous variables.

Number and percentage for categorical, and mean and standard deviation (SD) for continuous variables was calculated. Descriptive statistics for characteristics of study participants were calculated. We also compared micronutrient deficiency among anemic and non-anemic children. Statistical analysis was performed using SPSS statistical software version 24 [38].

We had compared median, Interquartile Range (IQR) of all micronutrient levels among the performance categories of four cognitive tests, to see the difference in the median for the performance category of cognitive tests. We used Kruskal–Wallis test to see the statistically significance difference among various performance categories of cognitive tests.

Chi- square test was performed for the comparison of different level of micronutrient deficiency across the performance categories of four cognitive test. Odds ratio (OR) with 95% confidence interval (CI) were calculated for association of micronutrient deficiency among four cognitive tests. Two tailed test was used to test the hypothesis; p value < 0.05 was taken as statistically significant. Logistics regression model was used to assess the association of borderline or dull normal among four cognitive tests at different level of micronutrient deficiency, controlling for gender, SES and anthropometric indicators. Adjusted OR with 95% CI are being reported for all cognitive tests.

For micronutrient deficiency, we have used only those cases where data of all eight micronutrients was available (n = 1918). For the further analysis, to test the association of cognitive function with micronutrient deficiency, micronutrient deficiency was categorized as no, any one and two or more micronutrient deficiency (≥ 2 MD).

## Results

**Fig 1:**
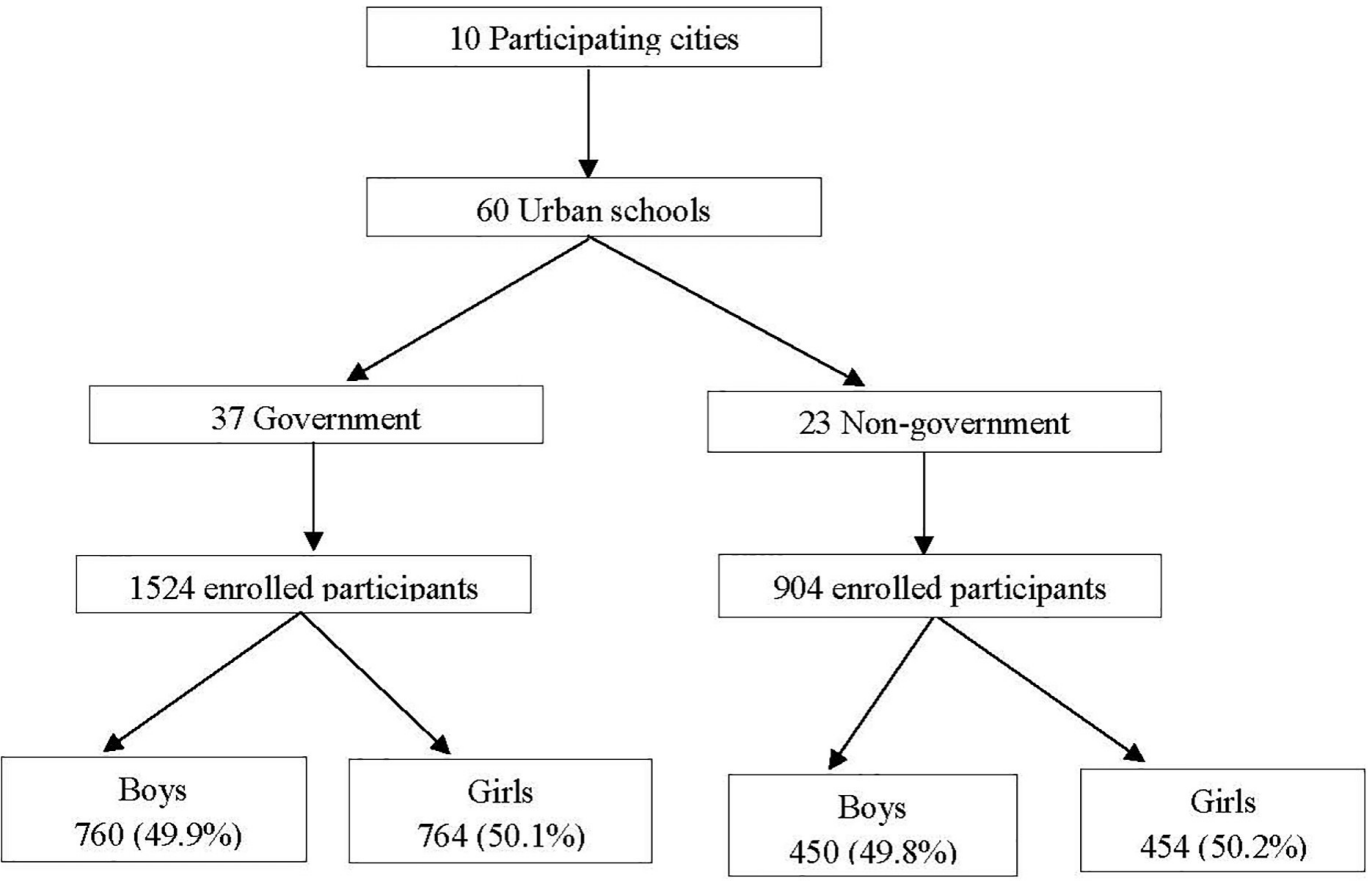
Flow diagram to show the selection process of schools and participants.

Among the study 2428 participants, 49.8% (1210/2428) boys were recruited from 60 schools. Of these, 37 (61.6%) from government funded and 23 (38.3%) from non-government. Selection process of schools and participants is shown in Fig 1.

Of the 2428 participants included, there were no difference in the number of family member, birth order, SES and anthropometric indicators among boys and girls (Table 2). Overall, only 7.0% (134/1918) participants had no deficiency. Two or more MD were more common in girls as compared to boys (73.4% vs 65.0%) [OR 1.45, 95% CI: 1.01–2.07, p = 0.04].

**Table 2:** Descriptive statistics for characteristics of study participants by gender.

Characteristics of variables	Overall	Boys	Girls
	m/n, row %		
*School Type*			
Government	1524/2428, 62.8	760/1524, 49.9	764/1524, 50.1
Private	904/2428, 37.2	450/904, 49.8	454/904, 50.2
*Number of family members*			
≤ 5 members	1547/2425, 63.8	807/1547, 52.2	740/1547, 47.8
> 5 members	878/2425, 36.2	400/878, 45.6	478/878, 54.4
*Birth order*			
First Child	1062/2425, 43.8	534/1062, 50.3	528/1062, 49.7
Second Child	851/2425, 35.1	424/851, 49.8	427/851, 50.2
Third or above	512/2425, 21.1	249/512, 48.6	263/512, 51.4
*Socio economic status*			
Upper or upper middle or lower middle	1403/2425, 57.9	717/1403, 51.1	686/1403, 48.9
Upper lower or lower	1022/2425, 42.1	490/1022, 47.9	532/1022, 52.1
*Anthropometric Indicators*			
Severe thinness or thinness	359/2428, 14.8	196/359, 54.6	163/359, 45.4
Normal	1682/2428, 69.3	829/1682, 49.3	853/1682, 50.7
Overweight	263/2428, 10.8	124/263, 47.1	139/263, 52.9
Obese or severely obese	124/2428, 5.1	61/124, 49.2	63/124, 50.8
*Micronutrient Deficiency*			
No deficiency	134/1918, 7.0	75/134, 56.0	59/134, 44.0
Any one deficiency	457/1918, 23.8	260/457, 56.9	197/457, 43.1
Two or more deficiency	1327/1918, 69.2	621/1327, 46.8	706/1327, 53.2

Comparison of micronutrient deficiencies and anemia by gender was shown in Table 3. Girls were found to be more deficient in vitamin D (62.1% vs 37.9%), iron (55.6% vs 44.4%), selenium (52.9% vs 47.1%) and folate (51.7% vs 48.3%) as compared to boys, whereas deficiency of zinc, vitamin A and vitamin B12 was lower in girls as compared to boys (Table 3). Girls were more anemic than boys (23.7% vs 11.6%) [OR 2.37, 95% CI: 1.90–2.95, p<0.001]. Comparing anemia with micronutrient deficiency, it was found that those with ≥ 2 MD were more common in anaemic children as compared to non-anaemic [OR 2.16, 95% CI: 1.22–3.81].

**Table 3:** Distribution of micronutrient deficiencies and anemia by gender.

Micronutrient	Overall	Boys	Girls
	m/n, %		
Calcium	1358/2267, 59.9	669/1358, 49.3	689/1358, 50.7
Iron	1119/2265, 49.4	497/1119, 44.4	622/1119, 55.6
Zinc	138/2026, 6.8	82/138, 59.4	56/138, 40.6
Selenium	208/2003, 10.4	98/208, 47.1	110/208, 52.9
Vitamin A	37/2250, 1.6	19/37, 51.4	18/37, 48.6
25 Hydroxy Vitamin D	900/2268, 39.7	341/900, 37.9	559/900, 62.1
Folate	505/2276, 22.2	244/505, 48.3	261/505, 51.7
Vitamin B12	759/2275, 33.4	407/759, 53.6	352/759, 46.4
*Anemia status*			
No anemia	1985/2410, 82.4	1062/1201, 88.4	923/1209, 76.3
Anemia	425/2410, 17.6	139/1201, 11.6	286/1209, 23.7
*Haemoglobin level (in mg/dl)*			
*n*, *mean (SD)*	2410, 12.76 (1.27)	1201, 13.08 (1.20)	1209, 12.43 (1.24)

**Fig 2:**
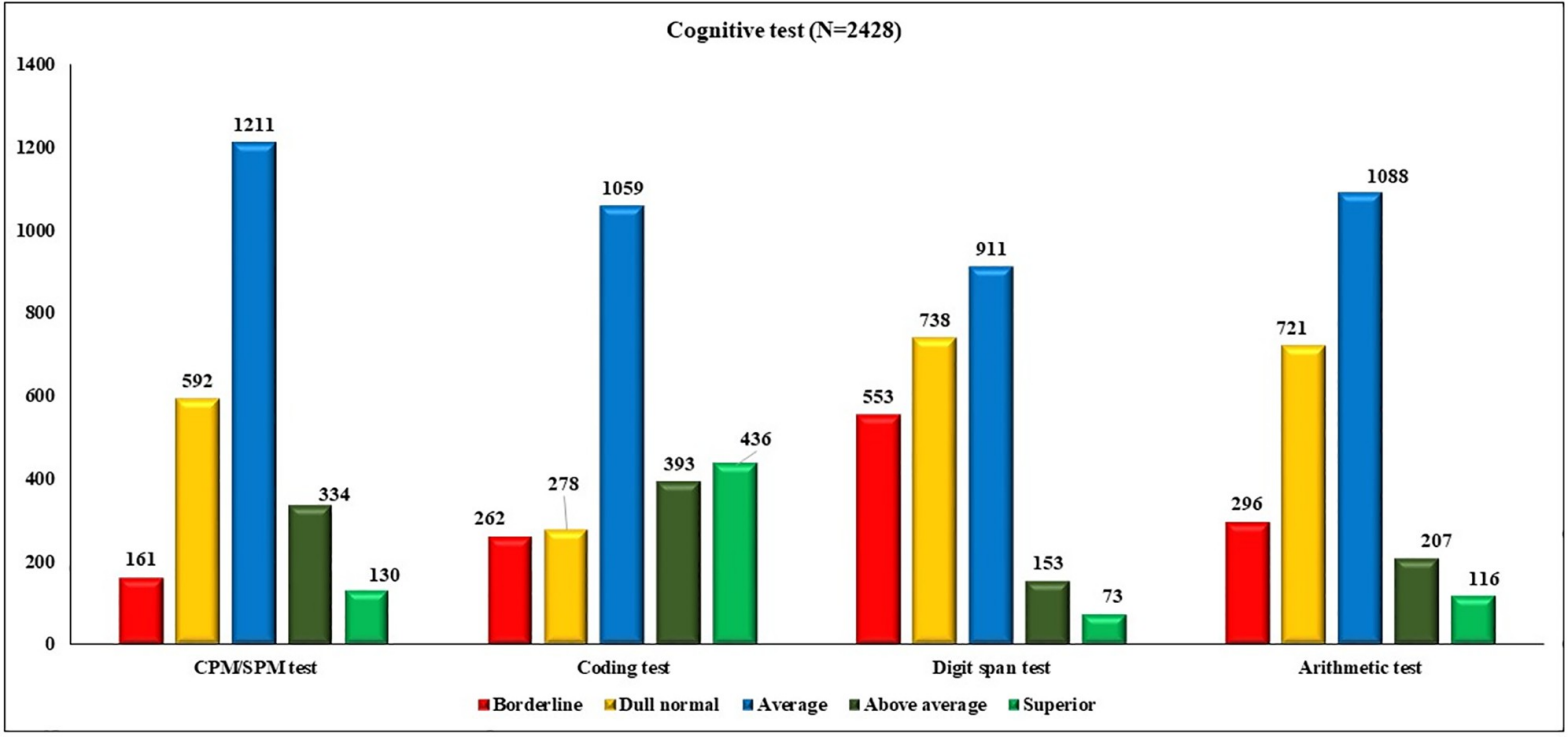
Distribution of participants on the basis of performance in various cognitive tests.

The distribution of participants on the basis of performance in various cognitive tests is shown in Fig 2. This shows that maximum participants are average performers followed by dull normal.

There was association of performance in various cognitive tests with level of micronutrients, as shown in S1 Table. We found that performance in cognitive tests improved with increasing levels of micronutrients. The association of different level of micronutrient deficiency with four cognitive tests was shown in S2 Table.

There was significant association of micronutrient deficiencies with CPM/SPM, Digit span and Arithmetic test of cognitive function (Table 4). Participants with ≥ 2 MD had increased odds of borderline or dull normal category for all the cognitive tests. The OR of ≥ 2 MD for CPM/SPM test was 1.66 (95% CI, 1.09 to 2.53), coding test was 1.61 (95% CI, 0.99 to 2.60), digit span test was 1.55 (95% CI, 1.09 to 2.22) and arithmetic test was 1.76 (95% CI, 1.20 to 2.57).

**Table 4:** Univariate association of different level of micronutrient deficiency with four cognitive tests.

Micronutrient Deficiency	Borderline or dull normal	Average or above average or superior	Odds Ratio	p value
m/n, %			(95% CI)	
**CPM/SPM test**				
*No deficiency	30/580, 5.2	104/1338, 7.8		**0.008**
Any one deficiency	121/580, 20.9	336/1338, 25.1	1.25 (0.79 to 1.97)	
Two or more deficiency	429/580, 74.0	898/1338, 67.1	1.66 (1.09 to 2.53)	
**Coding test**				
*No deficiency	21/424, 5.0	113/1494, 7.6		0.14
Any one deficiency	98/424, 23.1	359/1494, 24.0	1.47 (0.88 to 2.46)	
Two or more deficiency	305/424, 71.9	1022/1494, 68.4	1.61 (0.99 to 2.60)	
**Digit span test**				
*No deficiency	62/1049, 5.9	72/869, 8.3		**0.003**
Any one deficiency	228/1049, 21.7	229/869, 26.4	1.16 (0.79 to 1.70)	
Two or more deficiency	759/1049, 72.4	568/869, 65.4	1.55 (1.09 to 2.22)	
**Arithmetic test**				
*No deficiency	42/808, 5.2	92/1110, 8.3		**0.002**
Any one deficiency	175/808, 21.7	282/1110, 25.4	1.36 (0.90 to 2.05)	
Two or more deficiency	591/808, 73.1	736/1110, 66.3	1.76 (1.20 to 2.57)	

*Reference category are “No deficiency”

In logistic regression model (Table 5), controlling for gender, SES and anthropometric indicators, participants with ≥ 2 MD were found to be associated with borderline or dull normal performance category of cognitive tests. Those with ≥ 2 MD had increased adjusted odds of borderline or dull normal for CPM/SPM was 1.63 (95% CI: 1.05 to 2.52), coding was 1.66 (95% CI: 1.02 to 2.71), digit span was 1.55 (95% CI: 1.06 to 2.25) and arithmetic was 1.72 (95% CI: 1.17 to 2.53).

**Table 5:** Logistic regression model of different level of micronutrient deficiency with four cognitive tests controlling for sociodemographic variables.

Characteristics of variables	CPM/SPM test	Coding test	Digit span test	Arithmetic test
	Adjusted OR	Adjusted OR	Adjusted OR	Adjusted OR
	(95% CI)	(95% CI)	(95% CI)	(95% CI)
Micronutrient Deficiency*				
No deficiency				
Any one deficiency	1.27 (0.79 to 2.03)	1.47 (0.87 to 2.48)	1.14 (0.76 to 1.72)	1.36 (0.90 to 2.07)
Two or more deficiency	1.63 (1.05 to 2.52)	1.66 (1.02 to 2.71)	1.55 (1.06 to 2.25)	1.72 (1.17 to 2.53)
***Gender***	1.27 (1.04 to 1.56)	0.64 (0.51 to 0.79)	0.89 (0.73 to 1.07)	1.19 (0.99 to 1.43)
***Socio economic status***	2.54 (2.06 to 3.12)	2.12 (1.69 to 2.65)	3.10 (2.54 to 3.78)	1.77 (1.47 to 2.14)
***Anthropometric indicators*** ^***#***^				
Normal				
Severe thinness or thinness	1.41 (1.07 to 1.85)	1.22 (0.91 to 1.64)	1.06 (0.81 to 1.40)	1.12 (0.86 to 1.45)
Overweight or obese or severely obese	0.68 (0.49 to 0.94)	0.87 (0.62 to 1.22)	0.48 (0.37 to 0.64)	0.69 (0.53 to 0.91)

For micronutrients deficiency* ‘No deficiency’; and for Anthropometric indicators^#^ ‘Normal’, are taken as reference category.

## Discussion

The aim of the present study was to explore association of micronutrient deficiency with level of general intelligence, and specific cognitive functions, i.e., attention, concentration, visuomotor coordination and working memory, in urban school-going children and adolescents, aged 6 to 16 years, across ten cities of India.

We assessed eight micronutrients, of which four were minerals (calcium, iron, zinc and selenium) and four were vitamins (Vitamin A, vitamin B12, Vitamin D and folate). Micronutrients were assessed by standard acceptable methods in a single reference lab [20]. All quality control procedures were ensured. Cognitive function was assessed using CPM/SPM, coding, digit span and arithmetic tests. These are dependent on the age of child and are adapted for the Indian children. The tests were administered to the participants in individual sessions by trained psychologist and the results were documented.

We found that, borderline or dull normal performers in CPM/SPM test were 31%, in coding test were 22.2% and those in digit span and arithmetic tests were 53.2% and 41.9% respectively. Only 7.0% participants had no micronutrient deficiency, 23.8% had any one and 69.2% had ≥ 2 MD. It was found that, controlling for gender, SES and anthropometric indicators, ≥ 2 MD was associated with increased odds of borderline or dull normal for all cognitive function. All the micronutrients studied, had been associated with impaired cognitive function in various researches, either individually or in combination. There is also biological interaction of the micronutrients in cognitive functions, therefore to assess the impact of micronutrient deficiency on these, we categorized children into three categories: (a) no deficiency, (b) any one deficiency and (c) two or more deficiency.

We also found that attention and concentration abilities are significantly associated with all eight micronutrient levels we studied and the similar association is seen with general intelligence except for calcium. Visual-spatial abilities show association with calcium, folate, vitamin A and vitamin B12 levels and working memory was found to be associated with iron, folate, vitamin A and B12.

India is facing the challenge of micronutrient deficiencies, especially among children and adolescents. Anemia is a major public health problem in India. Our study demonstrates that 17.6% participants were anemic on basis of WHO defined criteria [28]. We found that anemia was associated with micronutrient deficiency. Since ≥ 2 MD were more common in anaemic children than non-anaemic (79.9% vs 67.0%, p<0.001), therefore anemia can be taken as surrogate marker. Studies have also reported that children with decreased hemoglobin levels were poor performers in cognitive tests [15]. Previous literature shows that iron, folate and vitamin B12 deficiencies are found to be associated with low scorings in cognitive tests [39–42], which is similar to the findings of current study.

In current study, we found that calcium, vitamin D and vitamin A levels are associated with cognitive performance, however probably no previous literature quoted findings related to these.

Systematic reviews on the impact of multi-micronutrient food fortification remains equivocal [3, 43, 44]. This variability in the literature may be explained by a number of factors, including differences in the formulation, study populations, contextual factors, and, notably, different methods used to assess cognitive performance [45].

The study was conducted as per the protocol published [20]. No modification or change were made while implementing the study.

The current research was a comprehensive multicentric work, with a good sample size, conducted to study the role of various crucial micronutrients in cognition including general intelligence as well as specific cognitive functions in school going children. However, this study was limited to urban settings only. Due to logistic issues, the sample size was equally divided across all study sites, this may have possibly under represented the sites with higher population densities. There could be a potential of bias introduced by unmeasured confounding factors in selection of participants. This is a cross-sectional study and hence there is no information about duration of exposure to various levels of micronutrients.

## Conclusion

All the micronutrients are related with the specific physiological processes which are accountable for cognitive and motor development in children. The current study demonstrates that two or more micronutrient deficiencies affect the cognitive outputs. These deficiencies are closely linked with developmental delays and low academic achievements. Henceforth effective strategies and plans such as nutritional diversification and food fortification are important for remodelling micronutrient intake in both developing and older children.

## Supporting information

S1 Table Distribution of performance of participants in various cognitive tests by micronutrient levels. (PDF)

S2 Table Association of different level of micronutrient deficiency with four cognitive tests. (PDF)
