# Supplementary material for: Micronutrients and cognitive functions among urban school-going children and adolescents: A cross-sectional multicentric study from India
Source: PLoS One. 2023 Feb 2;18(2):e0281247. doi: 10.1371/journal.pone.0281247 (PMC9894395; doi:10.1371/journal.pone.0281247)
Supplement: S1 Table — (PDF) [file pone.0281247.s001.pdf]

| S1 Table: Distribution of performance of participants in various cognitive tests by micronutrient levels | | | | | | |
|---|---|---|---|---|---|---|
| | Classification of cognitive performance | | | | | p-value |
| | Borderline | Dull normal | Average | Above-average | Superior | |
| **Levels of micronutrients [n, median (IQR)]** | | | | | | |
| *Calcium (mg/dl)* | | | | | | |
| CPM/SPM | 142, 10.0 (8.7-11.4) | 560, 9.7 (8.6-10.8) | 1114, 9.6 (8.7-10.6) | 318, 9.8 (9.0-10.6) | 128, 9.8 (8.9-10.3) | 0.12 |
| Coding Test | 237, 9.4 (8.0-10.4) | 263, 9.7 (8.6-10.8) | 984, 9.6 (8.7-10.7) | 372, 9.7 (8.9-10.7) | 406, 9.7 (8.9-10.7) | 0.02 |
| Digit Span | 524, 9.5 (8.1-10.5) | 703, 9.6 (8.6-10.5) | 828, 9.7 (8.9-10.9) | 141, 10.0 (9.3-11.0) | 66, 9.8 (8.8-11.8) | <0.001 |
| Arithmetic | 278, 9.8 (8.8-10.7) | 671, 9.6 (8.6-10.8) | 1016, 9.6 (8.7-10.6) | 191, 9.8 (8.9-11.2) | 106, 9.7 (8.7-10.7) | 0.20 |
| Iron (µg/dl) | | | | | | |
| CPM/SPM | 143, 65.3 (44.8-85.4) | 558, 69.2 (47.9-92.1) | 1117, 70.3 (51.0-92.0) | 318, 72.4 (51.6-93.9) | 127, 79.8 (57.4-99.3) | 0.02 |
| Coding Test | 237, 69.4 (48.8-92.0) | 262, 69.3 (47.2-87.2) | 986, 70.3 (51.1-94.6) | 372, 69.2 (48.4-90.8) | 406, 73.2 (53.3-93.8) | 0.28 |
| Digit Span | 523, 68.6 (45.0-88.0) | 705, 69.5 (51.1-92.1) | 826, 71.6 (50.8-95.0) | 142, 74.6 (55.9-98.7) | 67, 77.9 (53.8-95.0) | 0.04 |
| Arithmetic | 277, 67.1 (45.4-87.5) | 671, 69.1 (50.0-90.6) | 1017, 71.5 (51.1-94.0) | 193, 75.5 (53.8-96.0) | 105, 74.4 (49.0-95.8) | 0.04 |
| Zinc (µg/dl) | | | | | | |
| CPM/SPM | 114, 124.9 (97.7-158.0) | 499, 119.7 (94.2-157.8) | 1008, 116.7 (90.2-153.2) | 286, 118.9 (93.5-179.8) | 113, 127.5 (98.8-178.8) | 0.05 |
| Coding Test | 206, 117.3 (96.9-160.5) | 245, 119.5 (90.5-150.1) | 881, 118.4 (90.9-159.1) | 320, 120.3 (93.8-173.5) | 368, 118.3 (94.8-160.0) | 0.70 |
| Digit Span | 465, 125.5 (96.9-164.3) | 629, 117.9 (93.4-161.2) | 750, 116.1 (87.8-155.9) | 118, 111.9 (87.8-154.9) | 58, 116.1 (94.6-160.9) | 0.03 |
| Arithmetic | 242, 123.9 (95.7-159.7) | 605, 117.0 (91.1-159.1) | 902, 118.7 (93.6-159.0) | 172, 118.2 (90.2-160.2) | 99, 115.4 (91.0-174.1) | 0.84 |
| Selenium (µg/dl) | | | | | | |
| CPM/SPM | 113, 10.4 (7.9-14.5) | 495, 11.6 (8.8-15.7) | 997, 10.8 (7.6-14.9) | 285, 11.1 (8.6-15.2) | 113, 11.4 (8.4-17.5) | <0.001 |
| Coding Test | 205, 10.8 (8.5-15.7) | 242, 11.1 (7.9-15.7) | 870, 11.2 (8.1-15.5) | 316, 10.9 (7.9-14.2) | 370, 11.2 (8.4-14.8) | 0.56 |
| Digit Span | 463, 11.9 (8.9-16.3) | 624, 11.3 (8.4-15.4) | 741, 10.5 (7.6-14.5) | 117, 9.9 (6.9-13.4) | 58, 11.2 (8.8-15.5) | <0.001 |
| Arithmetic | 240, 11.2 (8.5-16.0) | 597, 11.0 (8.3-14.8) | 897, 11.1 (8.1-15.5) | 170, 11.0 (7.8-15.2) | 99, 10.4 (7.7-15.1) | 0.56 |
| *Vitamin A (µg/dl)* | | | | | | |
| CPM/SPM | 140, 48.8 (37.2-62.9) | 549, 53.6 (36.7-76.5) | 1116, 60.4 (42.2-89.6) | 319, 67.5 (48.0-106.6) | 126, 74.5 (56.6-123.8) | <0.001 |
| Coding Test | 235, 51.7 (36.7-82.3) | 258, 57.0 (41.5-88.8) | 984, 60.6 (41.6-88.6) | 365, 62.1 (45.1-94.7) | 408, 58.2 (42.7-88.8) | <0.001 |
| Digit Span | 510, 53.3 (35.8-80.5) | 702, 59.1 (40.7-89.9) | 829, 61.5 (45.3-91.3) | 140, 63.4 (46.2-92.2) | 69, 67.4 (47.7-94.8) | <0.001 |
| Arithmetic | 271, 58.7 (41.6-94.1) | 662, 58.3 (39.9-89.0) | 1016, 59.5 (41.3-86.1) | 196, 61.0 (43.4-90.3) | 105, 65.1 (47.6-101.1) | 0.23 |
| *Vitamin D* (ng/ml*)* | | | | | | |
| CPM/SPM | 143, 12.0 (8.4-17.6) | 558, 13.9 (10.1-19.4) | 1119, 13.6 (9.2-19.5) | 319, 13.9 (9.8-18.8) | 127, 15.7 (10.8-19.8) | 0.02 |

| | | | | | | |
|---|---|---|---|---|---|---|
| Coding Test | 238, 14.5 (9.8-20.6) | 264, 13.8 (9.5-19.5) | 987, 13.6 (9.6-19.3) | 369, 13.5 (9.6-18.0) | 408, 14.1 (10.0-18.7) | 0.47 |
| Digit Span | 523, 14.1 (10.0-19.6) | 708, 14.1 (9.9-20.1) | 825, 13.2 (9.2-18.2) | 142, 14.4 (9.7-19.1) | 68, 14.4 (10.4-18.1) | 0.03 |
| Arithmetic | 280, 13.0 (8.5-17.9) | 668, 13.6 (10.0-18.7) | 1019, 14.1 (9.6-19.5) | 192, 14.3 (10.4-19.2) | 107, 14.7 (9.8-20.1) | 0.09 |
| *Folate* (ng/ml) | | | | | | |
| CPM/SPM | 143, 3.8 (2.7-5.5) | 560, 4.2 (3.1-6.1) | 1124, 4.4 (3.2-6.2) | 322, 4.1 (2.9-6.0) | 127, 4.4 (3.3-5.9) | 0.01 |
| Coding Test | 240, 3.8 (2.8-5.5) | 264, 4.1 (2.8-5.7) | 992, 4.3 (3.2-6.1) | 370, 4.4 (3.2-6.4) | 410, 4.5 (3.2-6.5) | <0.001 |
| Digit Span | 525, 4.2 (2.9-6.1) | 710, 4.2 (3.1-6.0) | 831, 4.3 (3.2-6.1) | 141, 5.0 (3.5-6.6) | 69, 4.6 (3.1-5.7) | 0.03 |
| Arithmetic | 279, 3.9 (2.7-5.6) | 674, 4.2 (3.0-5.6) | 1020, 4.4 (3.2-6.4) | 196, 4.7 (3.1-7.1) | 107, 4.3 (3.3-6.5) | <0.001 |
| *Vitamin B12 (pg/ml)* | | | | | | |
| CPM/SPM | 142, 236 (174-341) | 558, 215 (153-315) | 1121, 257 (178-365) | 321, 303 (220-445) | 127, 392 (231-605) | <0.001 |
| Coding Test | 240, 233 (160-345) | 263, 242 (164-354) | 986, 249 (176-360) | 370, 285 (186-413) | 410, 272 (189-390) | <0.001 |
| Digit Span | 524, 221 (157.5-323) | 709, 257 (176-379) | 826, 275 (195-389) | 141, 274 (200-407) | 69, 242 (177-358) | <0.001 |
| Arithmetic | 278, 250 (177-350) | 673, 246 (162-354) | 1017, 252 (178-367) | 194, 282.5 (204-409) | 107, 326 (243-479) | <0.001 |
