# Supplementary material for: Micronutrients and cognitive functions among urban school-going children and adolescents: A cross-sectional multicentric study from India
Source: PLoS One. 2023 Feb 2;18(2):e0281247. doi: 10.1371/journal.pone.0281247 (PMC9894395; doi:10.1371/journal.pone.0281247)
Supplement: S2 Table — (PDF) [file pone.0281247.s002.pdf]

**S2 Table: Association of different level of micronutrient deficiency with four cognitive tests.**						
**Micronutrient Deficiency m/n, %**	**Borderline**	**Dull normal**	**Average**	**Above average**	**Superior**	**p value**
**CPM/SPM test**						
*No deficiency	4/134, 3.0	26/134, 19.4	71/134, 53.0	22/134, 16.4	11/134, 8.2	**0.03**
Any one deficiency	21/457, 4.6	100/457, 21.9	223/457, 48.8	80/457, 17.5	33/457, 7.2	
Two or more deficiency	81/1327, 6.1	348/1327, 26.2	659/1327, 49.7	172/1327, 13.0	67/1327, 5.0	
**Coding test**						
*No deficiency	8/134, 6.0	13/134,9.7	56/134, 41.8	23/134, 17.2	34/134,25.4	0.08
Any one deficiency	49/457, 10.7	49/457, 10.7	187/457, 40.9	73/457, 16.0	99/457, 21.7	
Two or more deficiency	136/1327, 10.2	169/1327, 12.7	594/1327, 44.8	208/1327, 15.7	220/1327, 16.6	
**Digit span test**						
*No deficiency	24/134, 17.9	38/134, 28.4	54/134, 40.3	14/134, 10.4	4/134, 3.0	**0.001**
Any one deficiency	85/457, 18.6	143/457, 31.3	173/457, 37.9	38/457, 8.3	18/457, 3.9	
Two or more deficiency	334/1327, 25.2	425/1327, 32.0	475/1327, 35.8	59/1327, 4.4	34/1327, 2.6	
**Arithmetic test**						
*No deficiency	9/134, 6.7	33/134, 24.6	78/134, 58.2	6/134, 4.5	8/134, 6.0	**0.005**
Any one deficiency	51/457, 11.2	124/457, 27.1	207/457, 45.3	50/457, 10.9	25/457, 5.5	
Two or more deficiency	174/1327, 13.1	417/1327, 31.4	572/1327, 43.1	104/1327, 7.8	60/1327, 4.5	
*****Reference category are "No deficiency"						
